# Fabrication of optimized oil–water separation devices through the targeted treatment of silica meshes

**DOI:** 10.1088/1468-6996/16/5/055006

**Published:** 2015-10-22

**Authors:** Colin R Crick, Feyza Tunali Ozkan, Ivan P Parkin

**Affiliations:** 1 Department of Chemistry, Imperial College London, South Kensington Campus, London SW7 2AZ, UK; 2 Materials Chemistry Research Centre, Department of Chemistry, University College London, 20 Gordon Street, London WC1H OAJ, UK

**Keywords:** superhydrophobic, oil–water separation, zeolite, surface functionalization, filter, water purification

## Abstract

Efficient oil–water separation is achieved using an optimized superhydrophobic material, generated by the zeolitic roughening and subsequent hydrophobic surface treatment of silica filter membranes. The material is both highly rough and intrinsically hydrophobic, resulting in superhydrophobic membranes which show a substantial affinity for hydrophobic solvents and oils. The membranes are syringe-mounted, suction pressure is applied and the selective collection of oil is achieved. The membranes are extremely robust, which is a result of the zeolitic roughening process, they possess small pores (0.7 *μ*m), as a result these devices can perform complete separation and operate at a range of suction pressures. The devices could be readily used in a range of real-world applications, including oil spill clean-up and industrial filters.

## 1. Introduction

The continual rise in the global demand for oil-based fuels will require the exploitation of new fossil fuel sources [1]. Untapped sources, which have been identified, may not be currently economically viable, due to their inaccessibility, size or other complexity. This is certain to change in the near future, as the depletion of current sources renders more arduous retrieval processes more attractive to oil companies. Given this inevitable rise in the risk of oil retrieval, the occurrence of environmental disasters such as the Deep Water Horizon (2010) spill may become more likely [2, 3]. This threat to the environment, global economic markets and the livelihood of people throughout the world, has been a huge drive behind the recent development of water purification technology [4, 5]. The development of materials able to selectively capture oils is an area of great interest to the companies involved in all aspects of oil retrieval, refinement and transport. Particularly with respect to the prevention and management of these major oceanic spill incidents [6]. An emphasis in the development of novel technologies is placed on their efficiency and ease of applicability.

The vast area of nanotechnology has been explored in order to tackle this scientific question, other areas focused on the development of nanomaterials include catalysis, transparent conducting films and biosensors [7–9]. Current state of the art devices utilize highly water repellent (superhydrophobic) materials which act to prevent the penetration of water, while allowing the free-flow of oils throughout the materials porosity [6, 10]. This has been demonstrated in the literature through membranes or filters that exhibit selective oil absorption, in addition to sponge-like materials that exclude water [11–15]. This technology can be readily achieved through the combination of porous materials (including ready-made filters and sponges) and hydrophobic coatings. Superhydrophobic materials have been widely explored in the literature, as well as having been exploited commercially [16–20]. The classification of these materials is generally made through examining the water contact angle, which is the angle made between the surface and the tangent made at the air–water surface interface by a water droplet lying on the surface. A water contact angle of above 150° is classed as superhydrophobic. This characterization has also been expressed through monitoring water bouncing on the surface [21, 22]. The fabrication methods used to generate these types of materials and surfaces is extensive, however superhydrophobic surfaces must have two main features. The first is a highly rough microstructure, which is exemplified by natural examples of superhydrophobicity such as the lotus plant (*Nelumbo nucifera*). The lotus leaves also demonstrate the second key property, which is an inherently water repellent coating, in this case the rough microstructure is coated with a hydrophobic wax [23]. Generally, the coatings can be substantial layers of material (mm or *μ*m), but even molecular monolayers have been utilize; so long as the surface roughness is maintained [16–20].

The use of pre-made filters or wools as substrates for generating oil–water separating materials has been previously reported in the literature [11–14]. A benefit of this approach is the filters or wools have inherent surface roughness, and could be rendered superhydrophobic with the addition of a hydrophobic surface coating. The most successful examples of this technology utilize filter membranes through which an oil–water mixture can be treated such that oil can pass through leaving water behind [24–27]. This approach has led to the development of several variants of near 100% efficiency filters, however these have also been accompanied with some inherent drawbacks [28, 29]. The major fault of many reported separation systems is the gravity feed aspect of them; the filters, once saturated with water, can no longer function to separate out a mixture as the less dense oil cannot make contact with the filter membrane. This is a particular issue when dealing with oils that are lighter than water, such as in the case of retrieving oil slicks on the surface of the ocean. Another issue with this type of separation system is caused by the gravity fed aspect of the devices, with the most effective systems that utilize smaller porosity require a greater amount of time for solvent to flow through the filter [30].

The material reported in this paper utilizes glass filter membranes, which are further roughened with a zeolitic reaction on the surface of the material. The material is then rendered superhydrophobic by a simple surface functionalization. The highly water repellent material was then utilized in fabricating an oil–water separation device. The devices not only show 100% separation efficiencies, but also overcome inherent disadvantages when using these systems. The syringe mounted membranes allow for a variation of suction pressures to be applied, able to be tuned to the oil being collected. This has the effect of greatly speeding up any separation relative to a gravity fed system, but the apparatus can also be readily repositioned to collect all oil from a mixture with no impedance of any water present. The zeolitic membrane reaction provides additional surface roughness, and imparts rigidity to the filters which allows for a higher suction pressure to be reached. The technology can be readily utilized for oil–water separation technology, both in oil spill clean-up and in industry.

## 2. Experimental details

### 2.1. Characterization techniques

X-ray diffraction (XRD) patterns were measured using a Bruker D4 diffractometer equipped with a Cu-K*α* radiation source (*λ* = 0.15418 nm). A scan range of 2°–50° with a step size if 0.02° was used, with a 4 s count time. Fourier transform infrared spectroscopy (FTIR) measurements were taken over a range of 400–4000 cm^−1^ using a Bruker Alpha FTIR with Opus software. Scanning electron microscopy (SEM) images were recorded using a Jeol 6700F FEG SEM operating at an acceleration voltage of 5 kV. Samples were vacuum sputtered with a thin film gold to improve surface conductivity within the SEM. Water contact angle measurements were carried out using an FTA-1000 drop shape instrument; 3 *μ*l water droplets were used and the contact angle of the water droplet was directly observed. Brunauer–Emmett–Teller (BET) surface area measurements were carried out using a Quantachrome Autosorb-IQ2 machine. Specific surface area was measured using the adsorption isotherm within relative pressures of 0.01 and 0.2, in accordance with the BET method. Each sample was weighed to ca 0.2 g and then degassed at 200 °C under vacuum for around 12 h before analysis. Tensile strength testing was carried out using a variable load apparatus, with load applied to the planar axis of the filters largest surface.

### 2.2. Materials

Glass microfibre filters, Fisherbrand MF 300, were purchased from Fisher Scientific. Motor oil (Carlube 5w30 Engine Oil) was purchased directly from the manufacturer. Ecoflex 5 silicone rubber was purchased from Bentley advanced materials. The remainder of the chemicals used in this investigation were purchased from Sigma-Aldrich chemical Co. including; hexamethyldisilazane (HMDS) (≥99.0%), tetraethylorthosilicate (TEOS) (≥99.0%), tetrapropylammonium hydroxide (TPAOH, 20% in water), petroleum ether (40 °C–60 °C, ≥95.0%), hexane (≥95.0%), chloroform (≥99.0%), toluene (≥99.5%), hydrogen peroxide (H_2_O_2_) (30% w/w in H_2_O), sulphuric acid (95%), methylene blue (≥82%) and oil red O (≥75%).

### 2.3. Zeolite transformation

The reaction of the amorphous silica filters to form silicalite-1 zeolite crystals was carried out under hydrothermal conditions. Silica samples were encased in a polytetrafluoroethylene lined stainless steel autoclave. The ideal reaction conditions used a reaction temperature of 130 °C for 6 h, the reactants added to the autoclave included TEOS, TPAOH and water in a 3:1:56 molar ratio. The ratio of reactants was not varied, however the length of reaction and reactor temperature was varied.

### 2.4. Hydrophobic surface treatment

Wool samples were placed in piranha solution for 10 min before being removed, rinsed and dried. The piranha solution was prepared by mixing one part aqueous hydrogen peroxide with three parts sulphuric acid. The resulting mixture was handled with extreme caution as piranha solution is strongly acidic and oxidizing. A solution of HMDS (10% v/v in toluene) was prepared and heated to 40 °C before adding the wool samples. These were then left in the solution for 24 h before being removed, rinsed with toluene and subsequently air dried.

### 2.5. Hydrophobicity measurements

Static water contact angles images were obtained using an FTA 1000B automated drop shape analyser using 3 *μ*l water droplets, surface baselines and subsequent tangents at the point of droplet contact were assigned manually to prevent error caused by image analysis software. For surfaces where averages were taken, a number of measurements were made across the films and the average values taken (average ten measurements).

### 2.6. Water–oil separation

**Figure 1.**
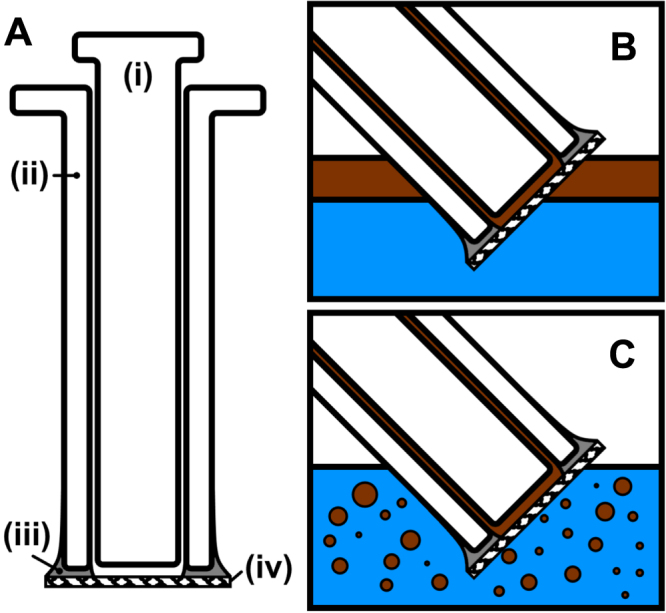
Illustration showing device schematic and oil collection methods. (A) Device comprises of (i) glass syringe plunger, (ii) shortened glass syringe barrel (needle adaptor removed), (iii) silicone adhesive and (iv) superhydrophobic silica membrane. The devices were used to collect a range of oils from water, the oil was collected as (B) a settled layer and (C) dispersed oil droplets.

Individual silica membranes were used to measure the absorption of each sample with respect to water and a range of oils (petroleum ether (40 °C–60 °C), toluene, hexane, chloroform and motor oil (Carlube 5w30 Engine Oil)). A glass syringe was cut horizontally at its tip, and the filter membranes were attached to the end of the syringe using an addition cured silicone rubber (Ecoflex 5), the filters were made secure by the use of additional hydrophobic rubber (figure 1). This ensured a secure fit to the pipette and also acted as a hydrophobic seal surrounding the edges of the membrane. Primarily the superhydrophobic filters were tested with pure oil, submerging the filter into the solvent and applying suction pressure to the syringe, after which mixtures of oil and water were used. This was repeated with deionized water. The selective removal of oil was achieved by orientating the filter at a tangent to the oil–water interface (figure 1). Vigorous agitation of the oil–water mixtures was used to form dispersed mixtures which were used in separation experiments (see supplementary information—S1). The filtered liquid was collected from the syringe and subsequently inspected.

## 3. Results and discussion

**Figure 2.**
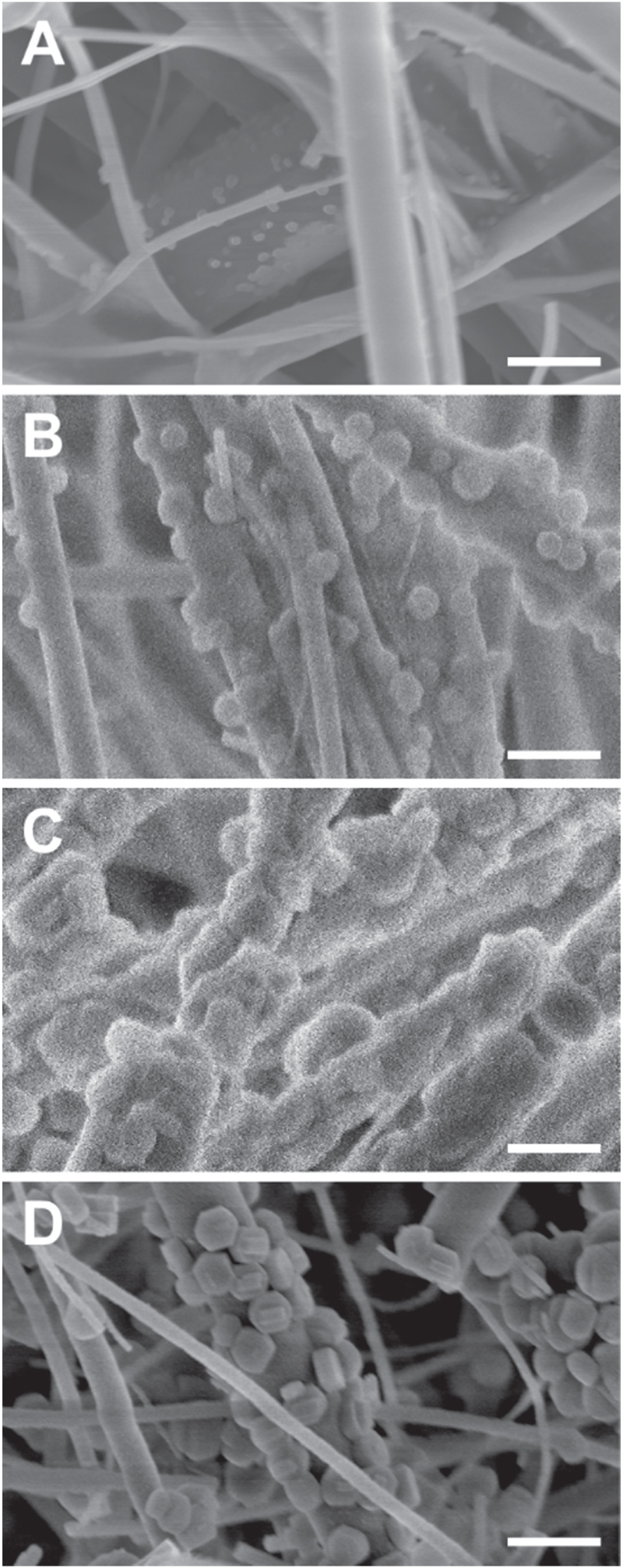
SEM images of the converted silica wool. The silica membranes were subjected to (A) 1 h, (B) 3 h, (C) 6 h and (D) 24 h of hydrothermal treatment. There is an observed increase in crystal growth as reaction time was increased. Samples subjected to 24 h of hydrothermal treatment showed the largest amount of silicalite-1 crystals, however the membranes lost their rigidity as they were transformed into a powder. Scale bar for all images shows 1 *μ*m.

The hydrothermal treatment of the amorphous silica to form silicalite-1 crystals on the meshes was carried out over a range of reaction times (1, 3, 6 and 24 h). These hydrothermal reactions were carried out in the presence of additional silica precursor (TEOS), the aim of this was to drive the formation of zeolite crystal on the fibres of the filter. The membranes physical appearance ranged from seemingly unchanged to undergoing a transformation into powder form as reaction time was increased. The micro/nanostructure of the mesh was characterized using SEM at each reaction time. The amount of silicalite-1 growth on the amorphous silica filter material is observed to increase with reaction time (figure 2). This was confirmed by examination of the crystalline structure via XRD (supplementary information—S2). The reaction time of 6 h was chosen as optimal, as the mesh was still intact and able to function as a filter, while also exhibiting a high surface roughness. The features added to the silica strands had an average diameter of 0.6 (±0.3) *μ*m and were 0.2 (±0.1) *μ*m thick.

The formation of the microporous framework of the silicalite-1 zeolite is facilitated by the silicon present in the TEOS precursor and silica membranes. The crystalline zeolite products, which contain of Si–O–Si linkages, are formed in hydrothermal conditions, facilitated by a ‘mineralizing agent’ (in this case TPAOH) [31]. SEM analysis (figure 2) revealed that the amount of silicalite-1 crystalline formed on the surface of the fibres was directly related to the hydrothermal exposure time. Higher coverage and size of silicalite-1 crystals was observed at longer hydrothermal times. The angular shape of the fully developed crystals (figure 2(D)) are caused by the crystalline structure of the silicalite-1 and the preferred crystal plane growth [32]. Further analysis of the images was carried out to monitor fibre shrinkage, however, no significant change was observed. This suggests that the only silica source consumed in hydrothermal reactions was from the precursor gel (TEOS). Indicating that the crystal growth occurs to the external surface of the fibres. This provides chemical binding between the filter fibre and the added crystals. BET measurements showed that the surface area increased from 6 to 91 m^2^ g^−1^ after a 6 h zeolitic treatment of the filters. This increase is a measurement of the additional roughness added by the zeolite crystals and is relatively small as the templating agent (TPAOH) was still present within the internal structure of the zeolite.

**Figure 3.**
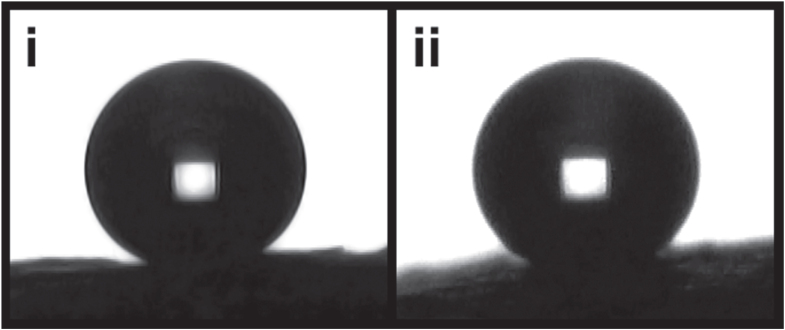
Water contact angles on the superhydrophobic silica wool with (i) no zeolitic transformation and (ii) silica filter exposed to a hydrothermal zeolitic treatment at a reaction temperature of 130 °C for 6 h. Both filters underwent a final step of hydrophobic surface functionalization with HMDS. The volume of the water droplets is 3 *μ*L, average water contact angles were measure at (i) 152° and (ii) 157°.

The ‘as received’ meshes were extremely hydrophilic, water contact angles could not be obtained as the water droplet was fully absorbed by the silica mesh. The zeolitic treatment also resulted in water droplets being completely absorbed by the mesh material. The two-step surface functionalization; piranha treatment to activate surface silanol groups followed by hydrophobic functionalization with HMDS, was carried out following the zeolitic transformation. There was no visible change or physical degradation of the meshes after each step of the surface functionalization. In addition, no distortion of nano/microstructure was observed via SEM. Functional group characterization using FTIR shows the addition of hydrophobic trimethylsilane [-Si-(CH_3_)_3_] groups (supplementary information—S3). Accurate quantification of the surface functionalization was not possible using infrared analysis, however literature reports estimate 40% of the surface groups are converted upon HMDS treatment [33, 34]. The meshes maintained their hydrophilicity upon the initial functionalization with piranha. The hydrophobic functionalization with HMDS resulted in the material becoming extremely hydrophobic. Water contact angles were measured at 157°, on the optimal meshes which underwent 6 h of hydrothermal treatment. This is higher than water contact angles measured on analogous silica meshes lacking zeolitic transformation, which were measured to be 152° (figure 3). These measured water contact angles are expected to be lower than those of the true value. This is a result of the macro-roughness of the filters (folds and bends in fibre that can be seen by eye), masking the true baseline of the substrate.

The ‘as received’ filters provided no selectivity of absorption and therefore no selectivity of separation. Filter membranes that were rendered superhydrophobic but with lack zeolitic transformation, provided effective separation for all oils. The oils used were allowed to pass through while water was completely excluded. A slow speed of extraction was required as the non-zeolitic membranes would fail if excess pressure was applied. An exception to this was the more viscous engine oil, which could not be separated as a high enough pressure could not be applied without breaching the membrane.

The superhydrophobic zeolitic membranes did not allow water to pass through even with a maximum suction pressure applied by the syringe. This was tested by using distilled water, which could not permeate the membrane. Full negative pressure was applied to the glass syringe, with no breeching of the membrane. This is estimated to be over−40 kPa, using a 5 mL syringe [35]. Tensile strength testing of the membranes before and after zeolitic functionalization showed that the materials gained a substantial tensile strength once transformed. The measurements were carried out by applying load along the longest axis of the membranes, this load was increase until the membranes were severed. The maximum weight supported by the ‘as received’ filters was 337 g, whereas the 6 h zeolitic treatment increased this to 375 g. Images detailing this can be seen in the supplementary information—S4. This added tensile strength goes toward explaining the higher resistance to the suction pressures experienced.

**Figure 4.**
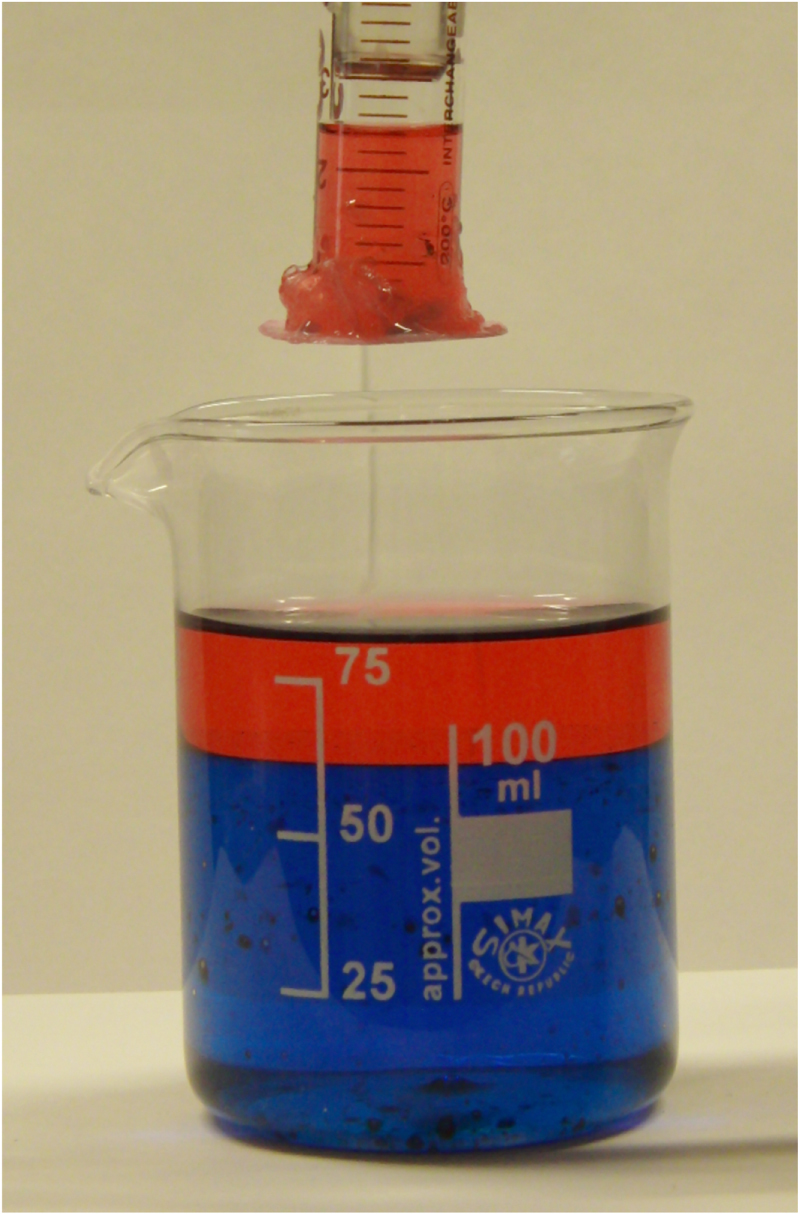
Image of the oil–water separation setup. The liquid, in this case water and petroleum ether, are dyed with methylene blue and oil red O respectively. The syringe contains the liquid collected from the interface, which is solely red in colour, indicating selective oil collection.

The membranes gave a 100% oil–water separation efficiency, even when applied to an emulsion created by vigorous agitation of the oil–water mixtures. This was validated by careful inspection of the liquid collected by the syringe, using a 20x optical microscope. Further experiments were carried out with dyes which aided visualization of each layer (figure 4). A video showing the selective oil collection is available in the supplementary information (S5). Collection of the more viscous motor oil was also possible as a higher suction pressure could be applied. The higher success of the superhydrophobic zeolitic membranes is readily justified. Primarily, the additional roughness, brought about by zeolitic crystal growth, promotes a higher surface hydrophobicity, this is reflected by the higher water contact angles achieved on these membranes. The growth of zeolite crystals had additional positive effects, including; an addition of rigidity, caused by partial agglomeration of membrane fibres (figure 2). The growth also caused a shrinking of the membranes pore size (closing by ∼0.2 *μ*m, ‘as received’ pores averaged 0.7 *μ*m), as zeolitic material was added to the surface of the fibres. This made it harder for of all liquids to pass through the membrane and a higher separation efficiency.

Membranes reported in the literature aimed at oil–water separation encompass a wide-range of materials which operate through an array of mechanisms. Although a substantial proportion of these reports detail near 100% separation efficiency, similar to that reported in this work, much of the reported research incorporates complex or costly fabrication techniques or material [36]. The key benefits of the membranes reported herein relate to its ease of fabrication, the inexpensive materials used, in addition to the material’s high potential for adaptability and further development in the future. An additional advantage is associated with the design of the separation device, allowing for the membrane to be directed toward oil contaminants for collection. Future development of the reported devices may include variation of the membrane thickness and micropore size in order to optimize the devices for a particular application.

## 4. Conclusions

The zeolitic membranes show complete oil–water separation. The devices can be applied to settled layers of oil or can be used to collect dispersed globules. The device construction and robust zeolitic material allow the disadvantages expressed in many existing reports to be surmounted. This is facilitated a device that is tuned toward oil capture, one that is not gravity feed and as a result can be directed to the oil contaminant, where the desired suction pressure can be applied. The membranes used in the study were selected as they possess small pores sizes (0.7 *μ*m), this was predicted to provide more effective separation of oil and water, as even small water droplets would also be repelled by the membrane. In future applications this could be varied in order to meet the requirements of designated applications, which may need a faster throughput of filtering. The devices can be readily used for oil–water separation applications, in a range of operational fields.
